# The TIR-NB-LRR pair *DSC1* and *WRKY19* contributes to basal immunity of Arabidopsis to the root-knot nematode *Meloidogyne incognita*

**DOI:** 10.1186/s12870-020-2285-x

**Published:** 2020-02-13

**Authors:** Sonja Warmerdam, Mark G. Sterken, Octavina C. A. Sukarta, Casper C. van Schaik, Marian E. P. Oortwijn, Jose L. Lozano-Torres, Jaap Bakker, Geert Smant, Aska Goverse

**Affiliations:** 1 Laboratory of Nematology, Wageningen University, Droevendaalsesteeg 1, 6708 PB Wageningen, The Netherlands; 2 Laboratory of Plant breeding, Wageningen University, Droevendaalsesteeg 1, 6708 PB Wageningen, The Netherlands

**Keywords:** *Meloidogyne incognita*, Arabidopsis, DSC1, WRKY19, Root-knot nematodes, TIR-NB-LRR receptor pair

## Abstract

**Background:**

Root-knot nematodes transform vascular host cells into permanent feeding structures to withdraw nutrients from the host plant. Ecotypes of *Arabidopsis thaliana* can display large quantitative variation in susceptibility to the root-knot nematode *Meloidogyne incognita*, which is thought to be independent of dominant major resistance genes. However, in an earlier genome-wide association study of the interaction between Arabidopsis and *M. incognita* we identified a quantitative trait locus harboring homologs of dominant resistance genes but with minor effect on susceptibility to the *M. incognita* population tested.

**Results:**

Here, we report on the characterization of two of these genes encoding the TIR-NB-LRR immune receptor DSC1 (DOMINANT SUPPRESSOR OF Camta 3 NUMBER 1) and the TIR-NB-LRR-WRKY-MAPx protein WRKY19 in nematode-infected Arabidopsis roots. Nematode infection studies and whole transcriptome analyses using the Arabidopsis mutants showed that *DSC1* and *WRKY19* co-regulate susceptibility of Arabidopsis to *M. incognita*.

**Conclusion:**

Given the head-to-head orientation of *DSC1* and *WRKY19* in the Arabidopsis genome our data suggests that both genes may function as a TIR-NB-LRR immune receptor pair. Unlike other TIR-NB-LRR pairs involved in dominant disease resistance in plants, DSC1 and WRKY19 most likely regulate basal levels of immunity to root-knot nematodes.

## Background

The root-knot nematode *Meloidogyne incognita* is currently ranked as one of the most invasive plant disease-causing agents, having major impact on global agricultural productivity [1]. Infective second stage juveniles (J2) of *M. incognita* penetrate their host at the root elongation zone. Thereafter, they migrate through the cortex to the root tip and enter the vascular cylinder via the columella and quiescence center. Inside the differentiating vascular cylinder, the J2 carefully puncture the cell walls of several host cells with their stylet to initiate the formation of a permanent feeding site [2–5]. This permanent feeding site includes several giant cells, which are formed by major structural and metabolic changes in host cells, most likely in response to stylet secretions of *M. incognita* [2, 6]. Juveniles of *M. incognita* take up their nutrients from these giant cells during the course of several weeks while undergoing three molts to enter the adult stage. Adult females produce eggs, which are held together in a gelatinous matrix at the surface of the roots [2–4].

Plants have developed several lines of defense to protect themselves against attacks by parasitic nematodes [3]. The first line of defense is thought to be structural, where plants make use of rigid cell walls to prevent host invasion (i.e., by migratory ectoparasites). Next, plant cells carry surface-localized receptors to detect molecular patterns in the apoplast that are uniquely associated with host invasion by endoparasitic nematodes [4, 7, 8]. For example, root-knot nematodes release small glycolipids commonly referred to as ascarosides that are recognized as invasion-associated molecular patterns [9]. The exposure of Arabidopsis seedlings to these ascarosides activates basal plant defenses to a broad range of pathogens. Furthermore, Arabidopsis mutant analyses (including *BRASSINOSTEROID INSENSITIVE 1 (BRI1)-associated receptor kinase 1 (BAK1)*) have shown that receptor-mediated basal immunity plays a significant role in the susceptibility of plants to root-knot nematodes [10]. Interestingly, root-knot nematodes have effectors capable of selectively suppressing responses activated by surface-localized immune receptors, which indicates adaptation to this line of defense [11–14].

At a later stage in the infection process, nematode resistant plants can counteract the establishment of a permanent feeding site with effector-triggered immunity, which is predominantly based on sensing nematode effectors by intracellular immune receptors [3, 15]. Effector-triggered immunity to root-knot nematodes often involves a hypersensitive-response in- and around giant cells, which interrupts the flows of assimilates towards the feeding nematodes. As a consequence of insufficient supply of nutrients, this type of major resistance induces a developmental arrest in juveniles.

The largest group of intracellular plant immune receptors is formed by the nucleotide-binding site leucine-rich repeat (NB-LRR) superfamily of immune receptors. These NB-LRRs consist of a central nucleotide-binding domain attached to a C-terminal leucine-rich repeat (LRR) domain and a variable N-terminal domain that can either be a coiled-coil (CC) or a Toll-interleukin-1 receptor (TIR)-like domain [4, 16–18]. Three major resistance (*R*) genes encoding intracellular NB-LRR immune receptors conferring resistance to *M. incognita* are cloned, one of which is encoding a CC-NB-LRR receptor (*Mi-1.2* from *Solanum peruvianum*) and two encode TIR-NB-LRR proteins (*Ma* from *Prunus cerasifera* and *PsoRPM2* from *Prunus sogdiana*) [19–21]. Most of the commercially grown tomato varieties (*Solanum lycopersicum*) carry introgressions of the *Mi-1.2* gene, conferring high levels of resistance to several tropical root-knot nematode species (e.g., *M. incognita, M. javanica* and *M. arenaria*) [22, 23]. Resistance based on *Mi-1.2* is currently losing efficacy in the field due to its temperature sensitivity and because of natural selection of virulent nematode populations [19, 24, 25]. The breakdown of *Mi-1.2* resistance and the small number of major resistance genes currently available for root-knot nematodes has prompted a search for alternative strategies to develop durable nematode resistant crops.

Previously, we used genome-wide association mapping to identify less conducive allelic variants of genes that critically determine susceptibility of Arabidopsis to *M. incognita* (i.e. *S*-genes [26, 27];). In total, we identified 19 QTL (quantitative trait loci) in the genome of Arabidopsis contributing to quantitative variation in reproductive success of *M. incognita*. We selected Arabidopsis as a model host for our studies because it is thought to lack major resistance to *M. incognita*. However, one of the QTL with minor effect on susceptibility of Arabidopsis to *M. incognita* harbors homologs of the TIR-NB-LRR class of resistance genes. Here, we report on the functional characterization of these TIR-NB-LRR genes that were previously annotated as *DSC1* and *WRKY19*. *DSC1* encodes a typical TIR-NB-LRR immune receptor [28], whereas *WRKY19* also includes a WRKY domain and MAPx domain at the C-terminus [29]. Our data from mutant analyses and whole transcriptome analyses suggest that the coordinated activity of DSC1 and WRKY19 as a receptor pair may be involved in regulating basal defense of Arabidopsis to *M. incognita*.

## Results

### Multiple TIR-NB-LRR protein encoding genes in a QTL for susceptibility

**Fig. 1:**
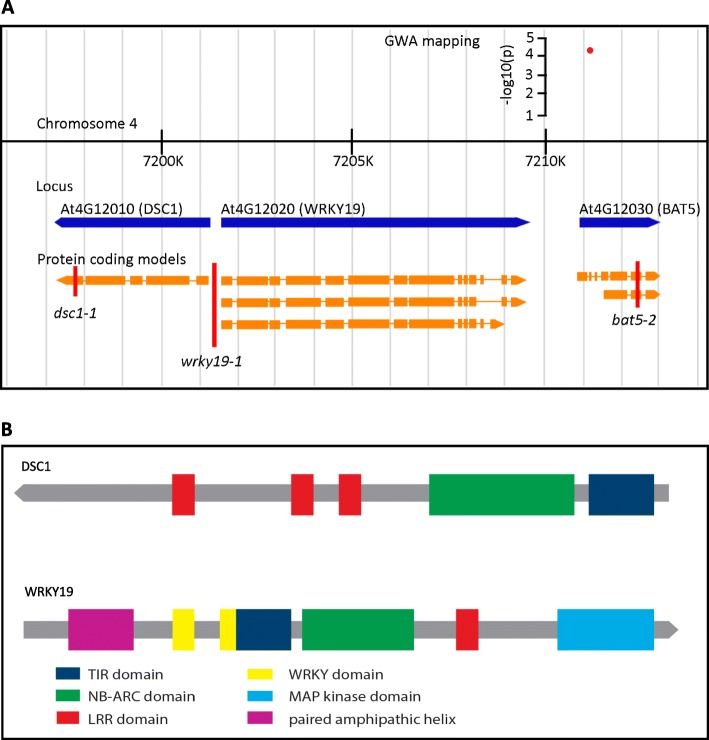
Genomic orientation of *BAT5*, *WRKY19* and *DSC1*, including the protein domains present in WKRY19 and DSC1. **a**, Representation of the genomic region around significantly associated SNP marker 138,422. The red dot represents the SNP with the corresponding –log10(p) score from the genome wide association mapping. The blue arrows represent the predicted genes. Transcripts derived from these genes are indicated in orange, with rectangles marking the protein coding exons. The red vertical line indicates a T-DNA insert with the corresponding name. **b**, Schematic representation of protein domains present in DSC1 and WRKY19. Colored blocks represent the different domains present in the protein sequence

In our previously published GWA (genome-wide association) study of the susceptibility of Arabidopsis to *M. incognita* we identified a single nucleotide polymorphism (SNP) marker on chromosome 4, which was closely linked to two genes with similarity to TIR-NB-LRR-type immune receptors [26]. This SNP marker (138442) was located inside an exon of *BILE ACID TRANSPORTER 5* (*BAT5*; At4G12030) and leads to a non-synonymous mutation (Val to Ala) close to the amino terminus of the protein (Fig. 1a). However, located directly upstream of *BAT5* are *DOMINANT SUPPRESSOR of Camta3 NUMBER 1* (DSC1; At4G12010) and *MITOGEN-ACTIVATED PROTEIN KINASE-WRKY19* (WRKY19; At4G12020), which both possess TIR, NB-ARC and LRR domains (Fig. 1a and b). *BAT5*, *DSC1* and *WRKY19* are all within 10 kb of SNP marker 138,442 and could therefore be causal for the effect on susceptibility to *M. incognita* associated with this marker.

### BAT5 is not causally linked to susceptibility of Arabidopsis to *M. incognita*

**Fig. 2:**
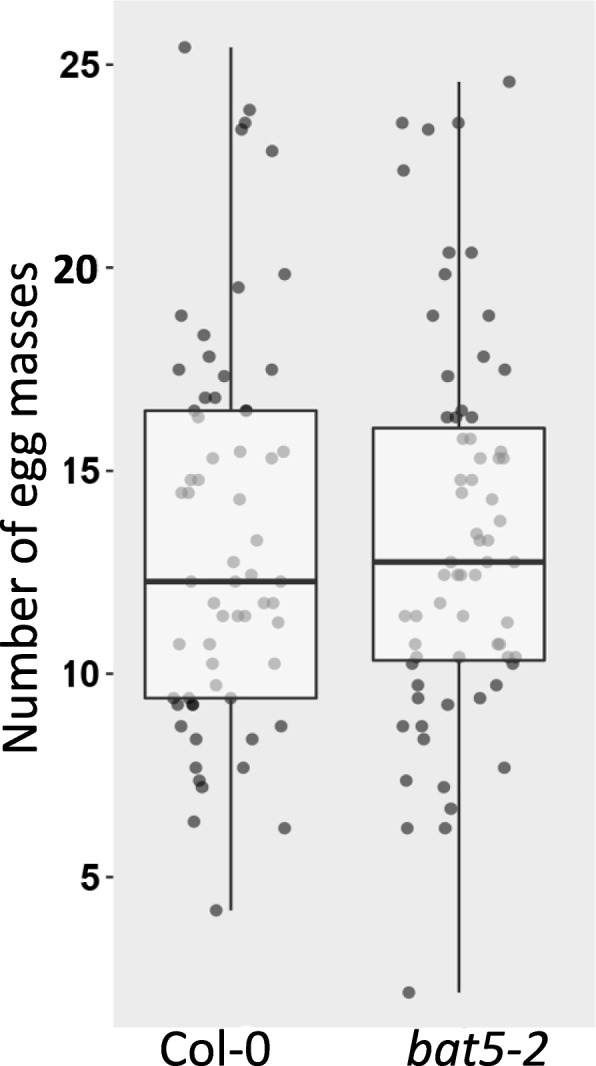
Susceptibility of the homozygous Arabidopsis T-DNA line *bat5–2* to *M. incognita*. Number of egg masses per plant at 6 weeks post inoculation on *bat5–2* and wild-type Arabidopsis Col-0 plants. Bars reflect the averages and standard error of the mean of three independent experiments (*n* > 18 per experiment). Data were statistically tested for significance with ANOVA with post-hoc Tukey test (*p* < 0.05)

Since SNP marker 138,442 is located inside the coding sequence of *BAT5*, we first tested if this gene is required for susceptibility of Arabidopsis to *M. incognita*. Thereto, we inoculated the roots of in vitro grown plants of the homozygous Arabidopsis T-DNA insert line *bat5–2* with infective J2 of *M. incognita*. The *bat5–2* mutant harbors a T-DNA insertion in the second last exon, resulting only in a slight reduction of mRNA levels of *BAT5* (Fig. 1a; Additional file 1). However, as the insert in *bat5–2* disrupts the open reading frame in *BAT5*, mRNAs are probably not translated into a functional protein. Nonetheless, six weeks after inoculation the number of egg masses produced by *M. incognita* per plant were not significantly different between *bat5–2* and wildtype Arabidopsis plants (Fig. 2). We also investigated the root architecture of the *bat5–2* mutant line at the time of inoculation (dpi = 0) as this may affect the susceptibility score of Arabidopsis for *M. incognita*, but we observed no significant difference in the number of root tips per plant for the *bat5–2* mutant compared to the wildtype Arabidopsis control plant Col-0 (Additional file 2). Altogether, our results provide no evidence for a significant role for BAT5 in susceptibility of Arabidopsis to *M. incognita*.

### *DSC1* and *WRKY19* may both regulate susceptibility of Arabidopsis to *M. incognita*

**Fig. 3:**
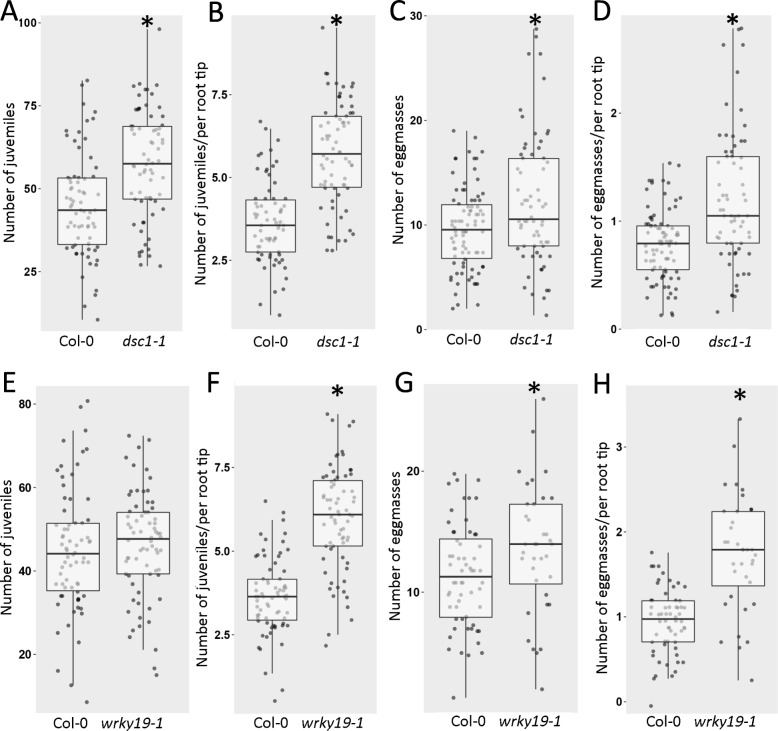
Susceptibility of homozygous Arabidopsis T-DNA lines *dsc1–1* and *wrky19–1* to *M. incognita*. **a, e** number of parasitic juveniles per plant at 7 days post inoculation (dpi = 7) on *dsc1–1, wkry19–1* and wildtype Arabidopsis Col-0 plants. **b, f** number of parasitic juveniles per plant at 7 days post inoculation on *dsc1–1, wkry19–1* and wildtype Arabidopsis Col-0 plants corrected for the number of root tips at the start of the infection (dpi = 0). **c, g** Number of egg masses per plant at 6 weeks post inoculation on *dsc1–1, wrky19–1* and wildtype Arabidopsis Col-0 plants. **d, h** Number of egg masses per plant at 6 weeks post inoculation on *dsc1–1, wkry19–1* and wildtype Arabidopsis Col-0 plants corrected for the number of root tips at the start of the infection. Boxplot represent data of three independent experiments (*n* > 12 per experiment). Data were statistically tested for significance with ANOVA with post-hoc Tukey HSD (* *p* < 0.05)

To study whether *DSC1* and *WRKY19* were involved in susceptibility of Arabidopsis to *M. incognita*, we also tested the homozygous Arabidopsis T-DNA insertion lines *dsc1–1* and *wrky19–1* in nematode infection assays. The T-DNA insert in *dsc1–1* is located in the last exon of *DSC1*, which causes a complete knock-out of gene expression (Additional file 1). In contrast, the T-DNA insert in *wrky19–1* is located in the putative promotor regions of both genes, which leads to strong upregulation of *WRKY19* expression and a small but significant down-regulation of *DSC1* (Additional file 1). Susceptibility of the mutant and wildtype plants was tested at 7 days post inoculation in this study, which corresponds to an early stage in root-knot nematode parasitism that includes the successful invasion of the roots and the initiation of a proper feeding site for further development and reproduction (two hallmarks for host susceptibility to plant-parasitic nematodes). At seven days after inoculation with *M. incognita*, we observed a significantly higher number of juveniles inside roots of the *dsc1–1* mutant plants compared to the roots of wildtype Arabidopsis control plants (Fig. 3a). The number of juveniles inside the roots of the *wrky19–1* overexpressing mutant was also slightly, but not significantly, higher as compared to wildtype Arabidopsis control plants (Fig. 3e). However, it should be noted that we also observed a significant lower number of root tips per plant for both the *dsc1–1* and *wrky19–1* mutants compared to wildtype Arabidopsis plants at the time of inoculation (dpi = 0; Additional file 3). When we corrected our data for this difference in root architecture, the number of juveniles per root tip was significantly higher in roots of both *dsc1–1* and *wrky19–1* mutant lines as compared to wildtype Arabidopsis control plants (Fig. 3b and f). Likewise, at six weeks after inoculation the number of egg masses per plant root system and the number of egg masses per root tip was significantly higher in both the *dsc1–1* knock-out mutant line and *wrky19–1* overexpressing mutant line as compared to the wildtype Arabidopsis control plants (Fig. 3c, d, g, and f).

**Fig. 4:**
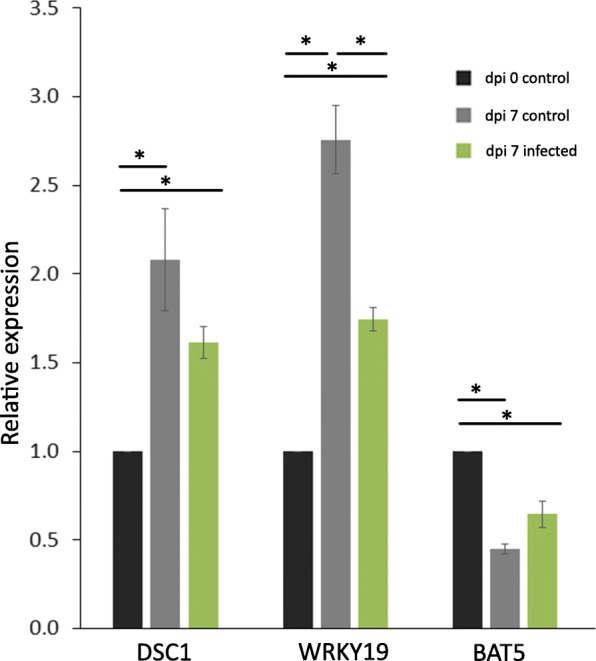
Relative expression of *DSC1, WRKY19* and *BAT5* in roots of Arabidopsis infected with and without *M. incognita*. Data is shown for whole roots collected at the time of inoculation with *M. incognita* (0 days control), for whole roots collected at 7 days after mock-inoculation (7 days control) and 7 days after inoculation with *M. incognita* (7 days infection). Data is represented as comparison against the expression level at 0 days control. Data is based on three independent experiments with three technical replicates per experiment. Error bars represent standard error of the mean. Data was analyzed with a student t-test (* = *p* < 0.05)

Our observations on the *wrky19–1* mutant line suggest that quantitative differences in expression levels of *DSC1* and *WRKY19* could influence both root development and susceptibility to *M. incognita* in opposite ways. We therefore investigated if the expression of *DSC1* and *WRKY19* is indeed regulated during root development and nematode infections in wildtype Arabidopsis plants using quantitative reverse transcription PCR (qRT-PCR) with gene specific primers (Fig. 4). Interestingly, both *DSC1* and *WRKY19* were upregulated in non-infected roots of Arabidopsis Col-0 when comparing 14 day old and 21 day old plants. This is consistent with a positive role for both genes in root development as suggested by the root phenotype of the *dsc1-1* and *wrky19–1* mutant lines. In contrast, we only observed a significant down-regulation of *WRKY19* in nematode-infected roots at 7 days post inoculation with *M. incognita*. This is consistent with a role for WRKY19 as a negative regulator of defense responses to *M. incognita*, as suggested by the increased number of nematodes on the *wrky19–1* mutant line, which is overexpressing *WRKY19*. Neither *DSC1* nor *BAT5* were significantly regulated in the roots of wildtype Arabidopsis Col-0 plants at seven days post inoculation with *M. incognita*. The lack of change in *DSC1* gene expression seems inconsistent with the observed increase in nematode infection on the *dsc1–1* mutant plants. However, this could indicate that no de novo synthesis of DSC1 is required for a role in regulating nematode susceptibility, or that we were not able to detect a local change in gene expression at the infection site due to the use of whole roots as input for the qRT-PCR analysis.

### *DSC1* and *WRKY19* regulate gene expression during *M. incognita* infection

**Fig. 5:**
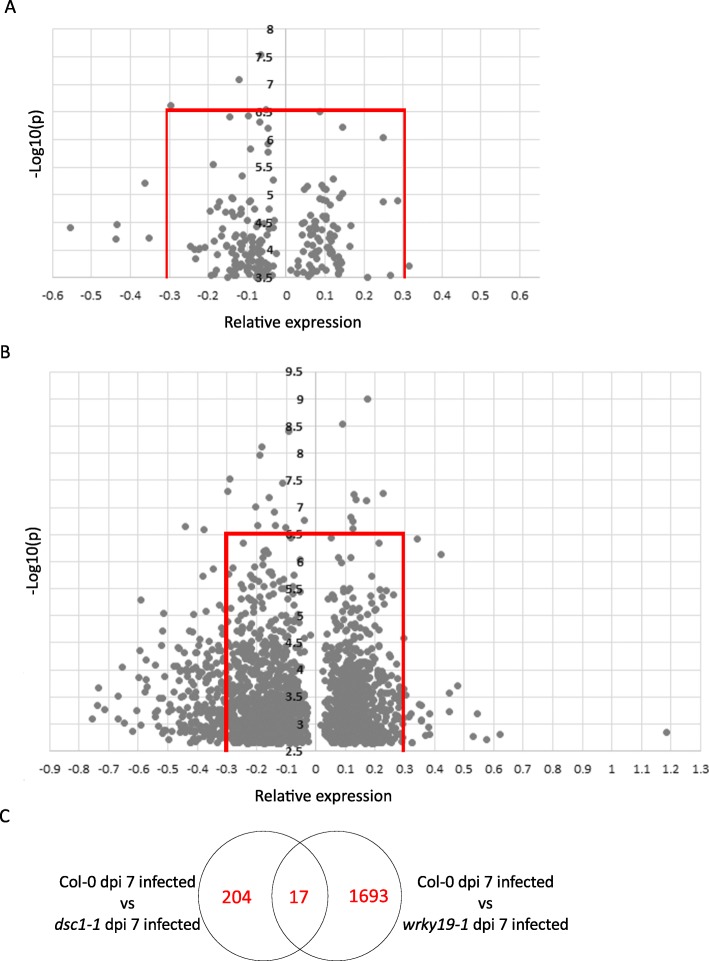
Differential expression analysis of *dsc1–1* and *wrky19–1* seven days after *M. incognita* infection. **a**, 221 genes were differentially expressed in *dsc1–1* compared to Col-0. The dot which represents the expression of *DSC1* with a significance of 10.5 and an effect size of − 2.50 is excluded from this figure for clarity. The red lines indicate the threshold for significance of 7 and effect size of 0.3. **b**, 1710 genes were differentially regulated in *wrky19–1* compared to Col-0. The red lines indicate the threshold for significance of 7 and effect size of 0.7. **c**, Venn diagram indicating the comparison between differently expressed genes in *dsc1–1* and *wrky19–1* 7 days after infection with *M. incognita*

**Fig. 6:**
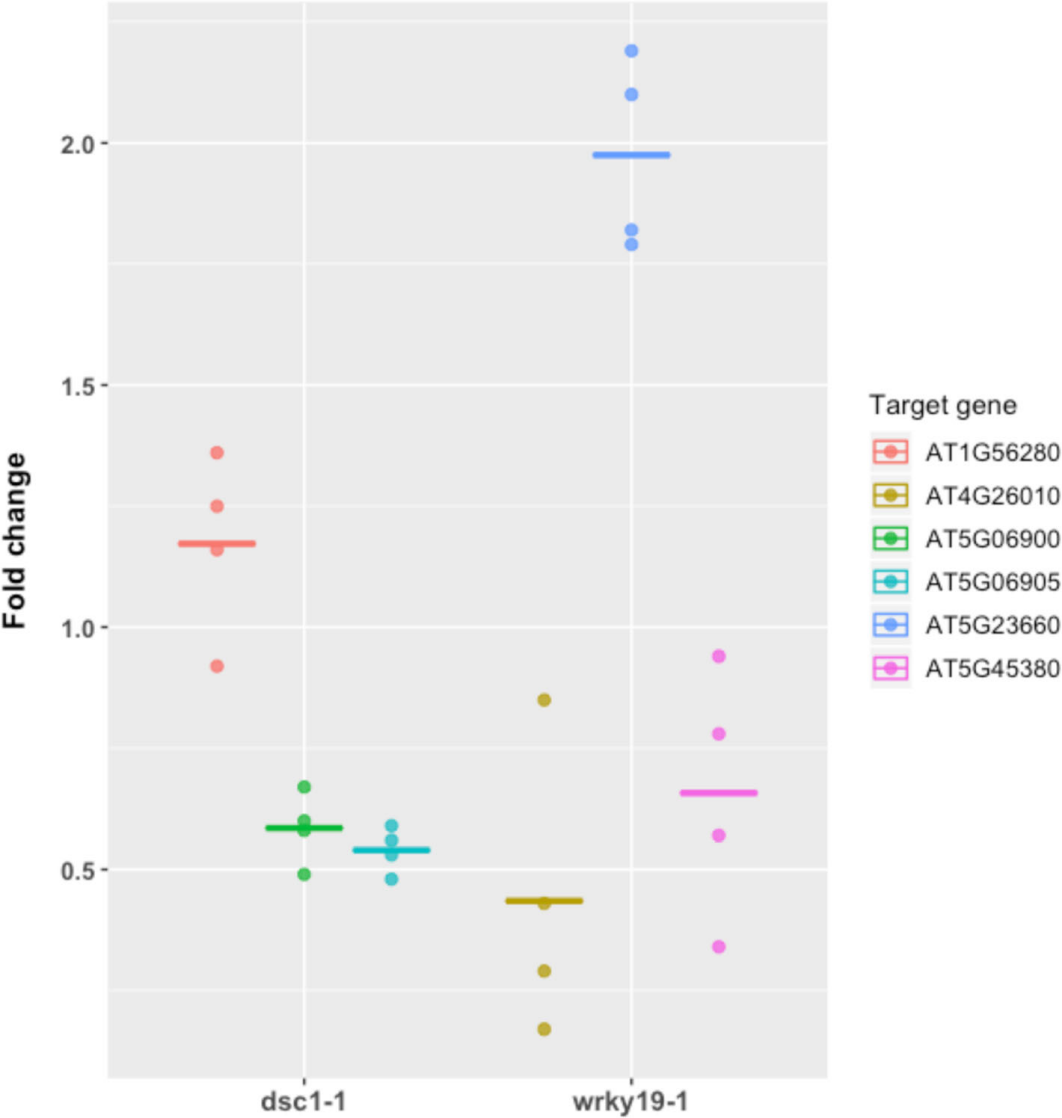
Relative expression of a selected set of genes identified in the microarray of *dsc1–1* or *wrky19–1* mutant plants (Table 1) relative to the wildtype Arabidopsis Col-0 background as determined by quantitative reverse-transcription PCR. Expression levels shown are represented as fold change measured in plants infected by *M. incognita* at the same time of inoculation (dpi =7). The data of each gene set consist of four biological replicates each comprising of three technical replications. Crossbar represents mean fold change

To gain more insight in the possible role of *DSC1* in susceptibility of Arabidopsis to *M. incognita*, we analyzed the transcriptome of whole roots of the *dsc1–1* mutant line and wildtype Arabidopsis control seven days after inoculation with *M. incognita* using Arabidopsis gene expression microarrays. As expected, in non-infected roots of the *dsc1–1* mutant expression of *DSC1* was absent, but – to our surprise – no other genes were differentially expressed in comparison with non-infected roots of wildtype Arabidopsis plants of the same age (at -log_10_(P) > 3.5). However, we observed the differential expression of 221 genes in a comparison between nematode-infected roots of the *dsc1–1* mutant and wildtype Arabidopsis control plants at seven days after inoculation (Fig. 5a). To determine which genes were strongly affected by the mutation in *dsc1–1*, we focused on genes that were either standing out because of a large change in expression level (i.e., mean normalized log_2_-change in probe intensities < − 0.3 or > 0.3), because of high statistical support of the changes (−log_10_(P) > 6.5)), or both (Fig. 5a). Applying these criteria resulted in a list of twelve differentially expressed genes in the absence of *DSC1*, which included several genes –like *DSC1* – with a link to abiotic- and biotic stress responses (Table 1, Fig. 5a). Three genes were selected for testing by qRT-PCR (Additional file 6) to confirm the observed changes in gene expression in the microarray analysis. Similar expression patterns were observed (Fig. 6), supporting the up- or downregulation of the selected genes in *M. incognita*-infected roots of the *dsc1–1* mutant at 7 dpi as observed in the microarray analysis.

**Table 1:** Differentially expressed genes in *dsc1–1* and *wrky19–1* at 7 days post inoculation with *M. incognita*

Gene ID	Significance	Relative expression	Gene description	WRKY domain in promotor	Associated with
Regulated in *dsc1–1*					
AT4G12010	10.71	−2.50	Dominant suppressor of CAMTA3 1 (DSC1)	N	Biotic stress response [28]
AT1G28040	7.55	−0.07	Ring/U-box superfamily protein	–	Putative ubiquitin ligase
AT2G38380	7.08	−0.12	Peroxidase superfamily protein	Y	Abiotic stress response [30]
AT4G16745	6.62	−0.29	Exostosin family protein	N	Pollen germination [31]
AT1G70360	6.54	− 0.05	F-box family protein	N	Putative ubiquitin ligase
AT5G57655	6.50	0.08	Xylose isomerase family protein	N	Recovery from abiotic stress [32]
AT3G52970	5.21	−0.36	Cytochrome P450, family 76, subfamily G, polypeptide 1	Y	
AT5G06905	4.46	−0.44	Cytochrome P450, family 93, subfamily D peptide 1	Y	Putative oxygen-binding activity
AT4G29690	4.41	−0.56	Alkaline-phosphatase-like family protein	Y	Hormone signaling and responses [33]
AT1G68040	4.22	−0.35	S-adenosyl-L-methionine-dependent methyltransferase superfamily protein	N	Defense [34]
AT5G06900	4.21	− 0.44	Cytochrome P450, family 712, subfamily A polypeptide 2	Y	Putative oxygen-binding activity
AT1G56280	3.72	0.32	drought-induced 19 (Di19)	N	Abiotic stress response [35]
Regulated in *wrky19–1*					
AT2G38330	9.00	0.18	Mate Efflux family protein	Y	Biotic stress response [36]
AT5G51860	8.53	0.09	K-box and MADS box transcription factor family protein (AGL72)	Y	Cell differentiation [37]
AT5G45380	8.41	−0.09	DEGRADATION OF UREA 3 (DUR3)	Y	Urea uptake [38]
AT1G34320	8.11	− 0.18	PSK SIMULATOR 1 (PSI1)	Y	Cell growth [39]
AT5G14470	7.96	−0.19	Galactokinase 2 (GALK2)	Y	Abiotic stress response [40]
AT1G64790	2.84	1.19	ILITHYIA (ILA)	Y	Biotic stress [41]
AT1G66870	2.80	0.62	Carbohydrate-binding X8 domain superfamily protein	Y	
AT3G48740	2.71	0.57	Sugars Will Eventually be Exported Transporter 11 (SWEET11)	Y	Biotic stress [42]
AT3G27940	3.13	0.54	LOB domain containing protein 26 (LBD26)	Y	Leaf development [43]
AT5G23660	2.77	0.53	Sugars Will Eventually be Exported Transporter 12 (SWEET 12)	N	Biotic stress [42]
AT4G26010	3.68	−0.73	Peroxidase super family protein	Y	Abiotic stress [44]
AT5G38910	3.34	−0.74	RmLC-like cupins superfamily protein	Y	Abiotic stress response [45]
AT3G47340	3.28	− 0.71	glutamine-dependent asparagine synthase 1 (ASN1)	Y	Metabolic pathways [46]
AT5G08250	3.09	−0.76	Cytochrome P450 superfamily protein	N	
At1G34510	3.09	−0.66	Peroxidase superfamily protein	Y	Wound response [47]

Gene expression of *dsc1–1* and *wrky19–1* is compared to Col-0. For each gene the level of significance and relative expression is stated, and the presence of a WRKY domain in the promotor region. The genes regulated in *dsc1–1* are ranked based on significance, whereas genes regulated in *wrky19–1* are presented in the following order: first the 5 most significant genes followed by the 5 genes most up-regulated and next, the 5 genes which are most down-regulated

To identify genes regulated in association with *WRKY19*, we also analyzed the transcriptome of whole roots of the *wrky19–1* mutant in non-infected and *M. incognita* infected plants. When comparing non-infected roots of the *wrky19–1* mutant and wildtype Arabidopsis control plants, no differentially expressed genes were observed (threshold for significance -log10(P) > 3.5). As expected, the expression of *WRKY19* was slightly – but not significantly – upregulated in the *wrky19–1* mutant line as compared to wildtype Arabidopsis (significance -log10(P) = 1.67; relative expression 0.22). However, in nematode-infected roots we detected 1710 differently expressed genes in a comparison between *wrky19–1* and wildtype Arabidopsis plants at 7 days after inoculation (Fig. 5b). Notably, the expression of DSC1 was significantly reduced in the *wrky19–1* mutant (significance -log10(P) = 5.2; relative expression − 0.31). To determine which genes are greatly affected in *wrky19–1,* we focused on genes that were above the threshold of significance of -log10(P) > 6.5 or had a relatively large change in relative expression (relative expression < − 0.3 or > 0.3). In total, 253 differentially expressed genes matched these criteria (Additional file 4). It was noted that 13 out of the 15 most significantly regulated genes - or those with largest relative expression of *wrky19–1* – contain a W-box motif ((T/A) TGAC(T/A)) in the corresponding promotor region (Table 1) consistent with a regulatory role for WRKY19 during nematode infection. Three genes from each category in Table 1 were selected for testing by qRT-PCR to confirm the observed changes in gene expression using the Arabidopsis microarray. Similar expression patterns were observed (Fig. 6), supporting the up or downregulation of the selected genes in *M. incognita-*infected roots of the *wrky19–1* mutant at 7 dpi. Most of the genes in this subset have been linked to biotic and abiotic stresses like observed for the *dsc1–1* mutant. For instance, the gene with by far the highest relative expression in *wrky19–1* is *ILITHYIA* (ILA, At1G64790), which encodes a HEAT-repeat protein required for basal defense to *Pseudomonas syringea* [41].

## Discussion

Previously, we mapped a QTL on chromosome 4 of Arabidopsis linked to reproductive success of *M. incognita* harboring two genes encoding TIR-NB-LRR proteins [26]. Although the SNP marker identified at this locus is located in *BAT5*, we did not find further evidence that allelic variation in this gene can be causal for variation in the number of *M. incognita* egg masses per plant at six weeks after inoculation (Fig. 2). Others have shown that BAT5 is associated with jasmonic acid-dependent signaling and wound responses [48], which are also relevant processes in the context of *M. incognita* infections [49–51]. Nevertheless, our data of the bioassays with the *bat5–2* knock-out mutant makes it unlikely that BAT5 plays a significant role in regulating the susceptibility of Arabidopsis to *M. incognita*.

After eliminating *BAT5* as a candidate susceptibility factor for *M. incognita* infections in Arabidopsis, we focused on the TIR-NB-LRR-encoding genes *DSC1* and *WRKY19* to explain the phenotypic variation associated with this locus. We show that the loss of *DSC1* expression in the *dsc1–1* Arabidopsis mutant significantly increases the number of juveniles per plant shortly after inoculation and the number of egg masses at the end of the life cycle of *M. incognita* (Fig. 3). This demonstrates that allelic variation linked to *DSC1* may indeed contribute to the phenotypic variation in the susceptibility of Arabidopsis to *M. incognita* [26].

Less straightforward is the interpretation of the data from our nematode infections assays with the *wrky19–1* Arabidopsis mutant. The T-DNA insert in *wrky19–1* is located 191 base pairs upstream of the transcription start site of *WRKY19* and 71 base pairs upstream of the transcription start site of the reversely oriented *DSC1* gene*.* Our qRT-PCR study suggested that the *wrky19–1* T-DNA insert decreases expression of *DSC1* but increases expression of *WRKY19* (Fig. S1). The microarray analysis shows also that *DSC1* expression is significantly reduced in roots of the *wrky19–1* mutant, but relative weakly. The expression of *WRKY19* is also increased in this mutant as expected, but not significantly (Fig. 5b). Although the transcriptional effects on either *DSC1*, *WRKY19* or both are mild in *wrky19–1*, we observe a significant phenotype in root architecture and susceptibility to *M. incognita* indicating that this mutation and the subsequent changes in *WRKY19* and *DSC1* expression has an impact on the condition of the plant.

We used transcriptome analyses by microarray to further investigate possible regulatory networks underlying the enhanced susceptibility of both *dsc1–1* and *wrky19–1* Arabidopsis mutants to *M. incognita*. Overall, we observe only a small overlap (17 genes) in the sets of differentially expressed genes in nematode-infected roots of *dsc1–1* and *wrky19–1* (Fig. 5c). Despite this small overlap in commonly regulated genes, we note that both sets are enriched for genes with a regulatory W-box in their putative promoter sequences (Table 1). The W-box is thought to be the consensus of the DNA binding site of WRKY domains of WRKY transcription factors [52]. The overrepresentation of the W-box could indicate that the regulation of these genes is indeed under control of the WRKY domain present in WRKY19. However, as we did not observe a major change in *WRKY19* expression in nematode-infected roots of the *wrky19–1* mutant as compared to wildtype Arabidopsis, this needs further investigation.

Another striking observation is the number of differentially expressed genes in nematode-infected roots of both *dsc1–1* and *wrky19–1* related to responses to abiotic stress (Table 1). This is in line with data from our earlier multi-trait genome wide association study showing that the susceptibility of Arabidopsis to *M. incognita* cross-correlates with responses to osmotic stress [53]. Likewise, we have recently shown that the transcription factor *ERF6,* which functions as a mediator of abiotic stress, is also required for susceptibility of Arabidopsis to *M. incognita* [26]. Altogether, these findings suggest that the ability to mitigate abiotic stress is one of the key regulating factors in susceptibility of the Arabidopsis to *M. incognita*.

The fact that many of the genes differentially regulated in the *dsc1–1* and *wrky19–1* mutants upon *M. incognita* infection have been linked to plant defense and responses to abiotic stress before might not be surprising. It is clear that nematode-infections are likely to cause stress in Arabidopsis roots. Furthermore, it has already been shown that DSC1 functions in plant immunity [28]. Nevertheless, to the best of our knowledge this is the first time that DSC1 can be linked to immunity to plant parasitic nematodes. DSC1 is a dominant suppressor of the calmodulin-binding transcription activator CAMTA3, which regulates resistance to various pathogens [54, 55]. The loss of DSC1 could increase the activity of CAMTA3 in nematode-infected roots of the *dsc1–1* mutant, leading to suppression of defense responses. However, no changes in *CAMTA3* expression was detected in the transcriptome data to support this model (data not shown). How DSC1 contributes to nematode defense needs further investigations.

Although, we cannot directly pinpoint the probable cause for the enhanced susceptibility of the *wrky19–1* mutant to *M. incognita*, our analyses of the transcriptome of nematode-infected roots of this mutant reveal a remarkably strong upregulation of the defense related gene *ILITYHIA* (*ILA*). *ILA* encodes a HEAT repeat protein, which is required for basal defense, resistance mediated by a subset of CC- and TIR-NB-LRR proteins, and systemic acquired resistance against *Pseudomonas syringae* [41]. *ILA* has not been linked to susceptibility of Arabidopsis to nematodes before, but the relative expression level of this gene in the microarray analyses is so high (relative expression of 1.19) that it could in fact be causal to the increased susceptibility of the *wrky19–1* mutant to *M. incognita*.

TIR-NB-LRR encoding genes can confer dominant disease resistance to Arabidopsis [56], but our data on the role of *DSC1* and *WRKY19* in nematode-infected roots does not point into that direction. First of all, the relatively low level of significance and small effect size of SNP marker 138,442 do not fit the typical dominant phenotype of a major resistance based on *TIR-NB-LRR* type *R* genes. Second, the differences in reproductive success of *M. incognita* on the *dsc1–1* knock-out mutant and the *wrky19–1* overexpressing mutant as compared to wildtype Arabidopsis plants were significant, but relatively small, and unlike major disease resistance responses conferred by *R* genes. We therefore conclude that *DSC1* and *WRKY19*, either separately or together as a pair, do not confer major resistance against *M. incognita* in Arabidopsis to the population tested. Instead, they are most likely involved in basal immunity to root-knot nematodes during early stages of the compatible interaction with Arabidopsis as a host plant.

A role for DSC1 and WRKY19 in basal immunity is consistent with observations by others that *DSC1* transcript levels increase upon application of SA (salicylic acid) or flg22 (flagellin 22) [57] and that *WRKY19* is thought to be an early component in regulatory networks of PTI [58]. Likewise, other TIR-NB-LRR proteins have been found to contribute to basal defense in Arabidopsis against *Pseudomonas syringae* (TN13) and the hemibiotrophic fungus *Leptosphaeria maculans* (RLM3) [59, 60]. Furthermore, the head-to-head genomic orientation of *DSC1* and *WKRY19* could indicate that they form an immune receptor pair [56, 57, 61]. So far, other *R*-gene pairs have been identified in Arabidopsis consisting of two tightly linked NB-LRR coding genes located in a similar head-to-head tandem arrangement [61]. For instance, the genomic organization of *DSC1* and *WRKY19* pair shows much similarity with that of the resistance to *Ralstonia solanacearum RRS1* and the resistance gene to *Pseudomonas* s*yringae RPS4* suggesting that they may act as a TIR-NB-LRR pair in plant immunity [56, 57, 61]. This is further supported by the similarities in protein architecture including the presence of functional domains required for immune receptor activity like the NB-ARC and LRR domain [62].

## Conclusion

Here, we provide first evidence for a functional role of *DCS1* and *WRKY19* in basal plant immunity to a plant pathogen as a *TIR-NB-LRR* gene pair. It will be interesting to investigate whether DSC1 and WRKY19 form indeed a functional protein complex and how this complex contributes to basal immunity in plants to root-knot nematodes.

## Methods

### Plant material

The following homozygous Arabidopsis T-DNA insertion mutant lines were obtained from the Nottingham *Arabidopsis* Stock Centre [63]: SALK_145043 with T-DNA insert in At4G12010 (*dsc1–1*); SALK_014300 with T-DNA in At4G12020 (*wrky19–1*); SALK_201408 with T-DNA in At4G12030 (*bat5–2*). The T-DNA insert lines were all generated in the background of Columbia-0 (Col-0 N60000), which was used as wildtype Arabidopsis throughout this study.

The presence and homozygosity of the T-DNA insert in the mutant lines was verified with PCR on genomic DNA isolated from leaf material of twelve seedlings [27]. The detection of the wild type allele or the T-DNA insert by PCR was performed as previous described [26] with different combination of primers for each line (Additional file 5) and the T-DNA-insert specific Lbl3.1 primer [63].

The expression of the T-DNA insertion affected gene was checked with reverse transcription quantitative PCR (qRT-PCR), the RNA was isolated as previous described [26]. To quantify the expression level for the gene of interest we used a gene specific forward and reverse primer (Table S1). For the qRT-PCR we used conditions as described below for gene expression analysis. Relative expression ratio between the gene of interest and the reference gene was calculated as described elsewhere [64].

### Nematode bioassay

Eggs of *Meloidogyne incognita* were obtained by treating tomato roots infected with *M. incognita* (strain ‘Morelos’ from INRA, Sophia Antipolis, France) as described [27].

To test the susceptibility of Arabidopsis seedlings, seeds were vapour sterilized and grown as described previously [27]. Individual seedlings were inoculated with 180 infective J2s of *M. incognita* per plant and incubated at 24 °C in the dark. The number of egg masses per plant were counted six weeks after inoculation by visually inspecting the roots with a dissection microscope. Each Arabidopsis genotype was tested in at least three independent experiments and 18 replicates per experiment. The obtained values were batch-corrected using the following equation: variable corrected = variable + (total mean (variable) - batch mean (variable)). The differences in counts per plants were statistically analysed using two-way analysis of variance (ANOVA) and post-hoc Tukey HSD test in R (version 3.0.2, www.r-project.org).

To collect roots of infected and non-infected Arabidopsis seedlings for gene expression analyses with microarrays and qRT-PCR, seeds were treated as described above for the susceptibility test. Seedlings were grown and inoculated and sampled as described [26].

### Root phenotype

The root phenotype of Arabidopsis seedlings was determined as described [26]. Differences in the total root length per seedling in cm and number of root tips were statistically analysed with a two-way ANOVA and post-hoc Tukey HSD test in R (*p* < 0.05).

### Gene expression analysis

Expression analysis for genes of interest was performed on the stored root samples produced during the nematode infection study. Whole root systems were cut from aerial parts of the seedlings and snap frozen in liquid nitrogen. Total RNA was isolated from whole roots of twelve 14-days-old plants of *dsc1–1*, *wrky19–1*, *bat5–2* and Col-0 wildtype. The RNA isolation and cDNA synthesis for quantitative reverse transcription PCR (qRT-PCR) from the roots was performed as described [26]. cDNA matching *Arabidopsis thaliana* elongation factor 1 alpha was amplified as a reference for constitutive expression using primers as indicated in Table S1 [65]. To quantify the expression level for the gene of interest specific gene primers were used (Table S1 & S2). The efficiencies of these primer sets were tested prior to the qRT-PCR analysis. For the qRT-PCR 5 ng cDNA was used with the following conditions: 15 min at 95 °C, forty cycles of 30 s at 95 °C, 30 s at 62 °C, and 30 s at 72 °C, and a final incubation of 5 min at 72 °C. Relative expression ratio between the gene of interest and the reference gene was calculated as described elsewhere [64]. Relative expression ratio was statistically analysed for significance and compared with a student t-test (*P*-value< 0.05).

For microarray analysis, approximately 200 ng of total RNA of each sample of Col-0, *dsc1–1* and *wrky19–1* were used for gene expression analysis on an *Arabidopsis* V4 Gene Expression Microarray (4x44K, Agilent Technologies). The microarray was performed as described i [26]. Two sets of data were generated: a set for comparison between Col-0 and *dsc1–1* and a set for the comparison between Col-0 and *wrky19–1*. The different sets for Col-0 contained different experimental samples. Each sample had at least three biological replicates.

### Microarray analysis

After scanning, the spot intensities of the microarrays were not background corrected [66]. Gene expression profiles were normalized using the Loess (within array normalization) and the quantile method (between array normalization) [67] in the Bioconductor Limma package [68]. The normalized intensities were log2-transformed for further analysis.

A linear model was used to identify differentially expressed genes in a side-by-side comparison. The following treatments were compared: day-0 control Col-0 versus mutant, day 7 control Col-0 versus mutant, day 7 infected Col-0 versus infected mutant, and day 7 control Col-0 versus infected Col-0. Each treatment consisted of three biological replicates. We used the linear model

$$ {\mathrm{E}}_{\mathrm{i}}={\mathrm{T}}_{\mathrm{i}}+\mathrm{error} $$

where the log2-normalized expression (E) of spot i (i in 1, 2, ..., 45,220) was explained over treatment (T). Afterwards, the obtained significances were corrected for multiple testing using the FDR procedure in p.adjust [69].

## Supplementary information

**Additional file 1: Figure S1.** Confirmation of T-DNA insert in line *bat5–2, dsc1–1 and wrky19–1* with PCR. A, allele specific PCRs on genomic DNA isolated from each Arabidopsis mutant line. PCR amplification products using primer combinations for only the wildtype gene allele (P1) and for the wildtype allele including the T-DNA insert (P2). B-D, Relative gene expression of the genes harbouring the T-DNA insert in the mutant lines as compared to the wildtype Arabidopsis Col-0 using quantitative RT-PCR on roots of 14-day old seedlings. B, represents the relative gene expression of *BAT5* in *bat5–2*. C, represents the relative gene expression of *DSC1* in *dsc1–1* and *wrky19–1*. D, represents the relative gene expression of *WKRY19* in *dsc1–1 and wrky19–1*. Data (B-D) was generated with three independent biological replicates with three technical replicates each.

**Additional file 2 **Number of root tips for *bat5–2* on 14-day-old seedlings. Statistically tested with ANOVA and post hoc Tukey test (*p* = 0.05). Data represents two biological replicates.

**Additional file 3 **The number of root tips for *dsc1–1* and *wrky19–1* on 14 day old seedlings. Statistically tested with ANOVA and post hoc Tukey test (*p* = 0.05); letters determine the group based on the level of significance. Data represents three biological replicates.

**Additional file 4 **245 regulated genes in *wrky19–1* during *M. incognita* infection with significance > 7 or with a relative expression of <− 0.3 or > 0.03.

**Additional file 5.** Overview of primers used in qRT-PCR and for confirmation of T-DNA insert.

**Additional file 6.** Overview of primers used in qRT-PCR for a set of selected genes in Table 1

## Data Availability

All microarray data were deposited in the ArrayExpress database at EMBL-EBI (www.ebi.ac.uk/arrayexpress) under accession number E-MTAB-6897. All other data generated during the current study are available from the corresponding author on a reasonable request.

## Abbreviations

ANOVA: Analysis of variance
BAK1: BRASSINOSTEROID INSENSITIVE 1 (BRI1)-associated receptor kinase 1
BAT5: BILE ACID TRANSPORTER 5
CAMTA3: Calmodulin-binding transcription activator number 3
CC: Coiled-coil domain
DSC1: DOMINANT SUPPRESSOR OF Camta 3 NUMBER 1 immune receptor
flg22: Flagellin 22
GWA: Genome-wide association
HEAT repeat: Acronym for a structural motif present in the four proteins Huntingtin, elongation factor 3 (EF3), protein phosphatase 2A (PP2A), and the yeast kinase TOR1
ILA: HEAT repeat protein ILITYHIA (after the Greek goddess of childbirth)
MAPx: MITOGEN-ACTIVATED PROTEIN KINASE
*Mi-1.2* gene: *Meloidogyne incognita* resistance gene number 1.2
NB-LRR: Nucleotide-binding site (NB) leucine-rich repeat (LRR) immune receptor
PCR: Polymerase Chain Reaction
qRT-PCR: Quantitative Reverse Transcription PCR
QTL: Quantitative Trait Locus
*R* gene: Resistance gene
RLM3: Resistance to *Leptosphaeria maculans* 3
RPS4: Resistance to *Pseudomonas syringae* 4
RRS1: Resistance to *Ralstonia solanacearum* 1
SA: Salicylic acid
*S*-gene: Susceptibility gene
SNP: Single Nucleotide Polymorphism
T-DNA: Transfer DNA
TIR: Toll-Interleukin-1 receptor (TIR)-like domain
TN13: TIR-NBS protein number 13
W-box: WRKY binding motif
WRKY: Conserved WRKY amino acid signature sequence
WRKY19: TIR-NB-LRR-WRKY-MAPx domain containing protein number 19
