# Supplementary material for: The TIR-NB-LRR pair DSC1 and WRKY19 contributes to basal immunity of Arabidopsis to the root-knot nematode Meloidogyne incognita
Source: BMC Plant Biol. 2020 Feb 13;20:73. doi: 10.1186/s12870-020-2285-x (PMC7020509; doi:10.1186/s12870-020-2285-x)
Supplement: Supplementary file 1 — Additional file 1: Figure S1. Confirmation of T-DNA insert in line bat5–2, dsc1–1 and wrky19–1 with PCR. A, allele specific PCRs on genomic DNA isolated from each Arabidopsis mutant line. PCR amplification products using primer combinations for only the wildtype gene allele (P1) and for the wildtype allele including the T-DNA insert (P2). B-D, Relative gene expression of the genes harbouring the T-DNA insert in the mutant lines as compared to the wildtype Arabidopsis Col-0 using quantitative RT-PCR on roots of 14-day old seedlings. B, represents the relative gene expression of BAT5 in bat5–2. C, represents the relative gene expression of DSC1 in dsc1–1 and wrky19–1. D, represents the relative gene expression of WKRY19 in dsc1–1 and wrky19–1. Data (B-D) was generated with three independent biological replicates with three technical replicates each. [file 12870_2020_2285_MOESM1_ESM.pdf]

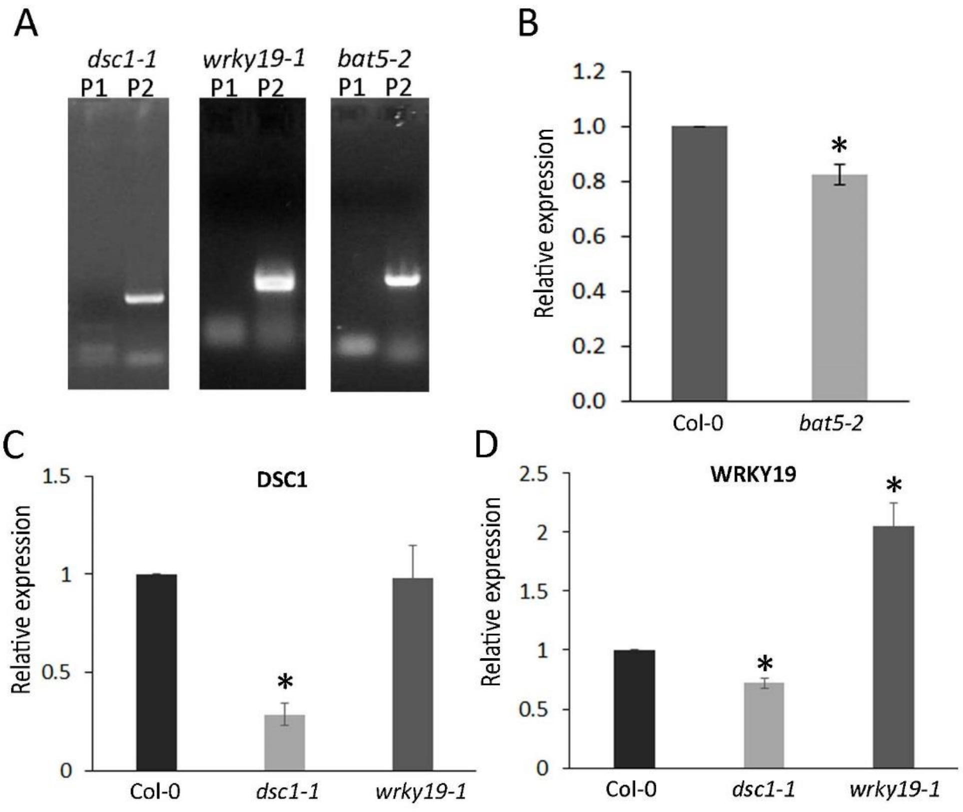

Additional file 1: Confirmation of T-DNA insert in line *bat5-2, dsc1-1 and wrky19-1* with PCR. A, allele specific PCRs on genomic DNA isolated from each Arabidopsis mutant line. PCR amplification products using primer combinations for only the wildtype gene allele (P1) and for the wildtype allele including the T-DNA insert (P2). B-D, Relative gene expression of the genes harbouring the T-DNA insert in the mutant lines as compared to the wildtype Arabidopsis Col-0 using quantitative RT-PCR on roots of 14-day old seedlings. B, represents the relative gene expression of *BAT5* in *bat5-2*. C, represents the relative gene expression of DSC1 in dsc1-1 and wrky19-1. D, represents the relative gene expression of WKRY19 in *dsc1-1 and wrky19-1*. Data (B-D) was generated with three independent biological replicates with three technical replicates each.
