# Supplementary material for: The TIR-NB-LRR pair DSC1 and WRKY19 contributes to basal immunity of Arabidopsis to the root-knot nematode Meloidogyne incognita
Source: BMC Plant Biol. 2020 Feb 13;20:73. doi: 10.1186/s12870-020-2285-x (PMC7020509; doi:10.1186/s12870-020-2285-x)
Supplement: Supplementary file 2 — Additional file 2 Number of root tips for bat5–2 on 14-day-old seedlings. Statistically tested with ANOVA and post hoc Tukey test (p = 0.05). Data represents two biological replicates. [file 12870_2020_2285_MOESM2_ESM.pdf]

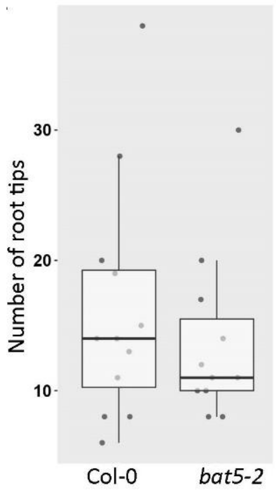

Additional file 2: Number of root tips for *bat5-2* on 14-day-old seedlings. Statistically tested with ANOVA and post hoc Tukey test (p= 0.05). Data represents two biological replicates.
