# Supplementary material for: The TIR-NB-LRR pair DSC1 and WRKY19 contributes to basal immunity of Arabidopsis to the root-knot nematode Meloidogyne incognita
Source: BMC Plant Biol. 2020 Feb 13;20:73. doi: 10.1186/s12870-020-2285-x (PMC7020509; doi:10.1186/s12870-020-2285-x)
Supplement: Supplementary file 3 — Additional file 3 The number of root tips for dsc1–1 and wrky19–1 on 14 day old seedlings. Statistically tested with ANOVA and post hoc Tukey test (p = 0.05); letters determine the group based on the level of significance. Data represents three biological replicates. [file 12870_2020_2285_MOESM3_ESM.pdf]

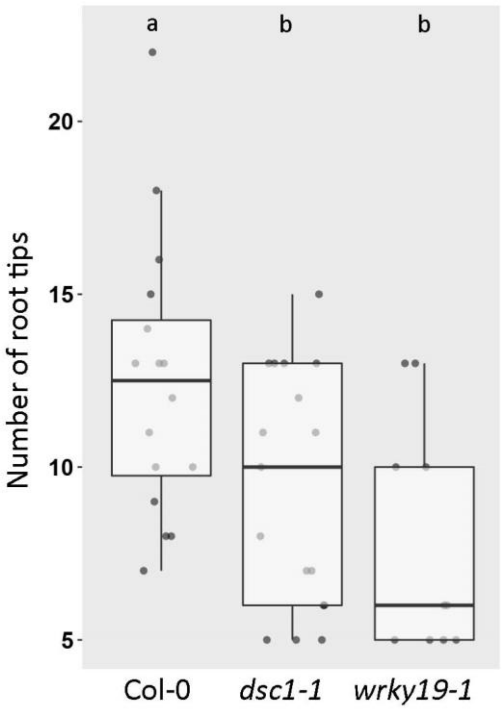

Additional file 3: The number of root tips for *dsc1-1* and *wrky19-1* on 14-day-old seedlings.

Statistically tested with ANOVA and post hoc Tukey test (P= 0.05); letters determine the group based on the level of significance. Data represents three biological replicates.
