# Supplementary material for: The TIR-NB-LRR pair DSC1 and WRKY19 contributes to basal immunity of Arabidopsis to the root-knot nematode Meloidogyne incognita
Source: BMC Plant Biol. 2020 Feb 13;20:73. doi: 10.1186/s12870-020-2285-x (PMC7020509; doi:10.1186/s12870-020-2285-x)
Supplement: Supplementary file 4 — Additional file 4 245 regulated genes in wrky19–1 during M. incognita infection with significance > 7 or with a relative expression of <− 0.3 or > 0.03. [file 12870_2020_2285_MOESM4_ESM.pdf]

Additional file 4: 245 regulated genes in *wrky19-1* during *M. incognita* infection with significance >7 or with a relative expression of <-0.3 or >0.3.

Gene ID	Significance	Relative expression	Gene ID	Significance	Relative expression
AT2G38330	9.00	0.18	AT3G45130	6.59	-0.38
AT5G51860	8.53	0.09	AT2G02300	3.33	-0.38
AT5G45380	8.41	-0.09	AT5G44550	3.37	-0.37
AT1G34320	8.11	-0.18	AT3G47480	3.77	-0.37
AT5G14470	7.96	-0.19	AT1G32940	4.13	-0.37
AT5G63730	7.53	-0.29	AT4G12550	3.76	-0.37
AT5G46470	7.45	-0.11	AT5G41040	5.08	-0.37
AT5G38320	7.30	-0.30	AT4G09100	3.84	-0.37
AT4G32890	7.25	0.23	AT5G48180	2.96	-0.37
AT4G36540	7.23	0.13	AT5G50200	4.07	-0.37
AT4G05020	7.18	-0.16	AT1G47890	4.06	-0.37
AT1G09770	7.15	0.13	AT1G05880	3.21	-0.37
AT1G12440	7.12	0.17	AT1G79330	2.85	-0.37
AT3G23840	7.02	-0.20	AT3G48640	4.31	-0.37
AT5G08250	3.09	-0.76	AT5G05500	3.83	-0.36
AT5G38910	3.34	-0.74	AT4G12545	3.85	-0.36
AT4G26010	3.68	-0.73	AT2G29620	3.13	-0.36
AT3G47340	3.28	-0.71	AT4G25790	4.53	-0.36
AT1G34510	3.09	-0.67	AT1G34050	3.20	-0.36
AT3G62680	3.51	-0.67	AT5G61010	3.50	-0.36
AT4G25820	4.05	-0.65	AT2G05910	3.26	-0.36
AT2G44925	3.02	-0.65	AT3G22565	4.21	-0.36
AT3G12700	2.86	-0.62	AT1G11190	2.78	-0.36
AT5G22555	3.26	-0.61	AT4G37290	3.90	-0.36
AT2G39010	3.87	-0.60	AT1G68450	3.12	-0.36
AT5G57625	4.36	-0.60	AT5G58840	3.61	-0.36
AT4G02270	2.98	-0.59	AT2G20825	2.71	-0.36
AT2G43580	5.29	-0.59	AT1G02575	2.69	-0.36
AT5G57625	3.59	-0.58	AT1G01560	3.20	-0.36
AT2G26560	4.19	-0.57	AT3G05155	4.05	-0.35
AT1G62980	3.81	-0.57	AT5G41570	2.74	-0.35
AT1G29020	3.68	-0.57	AT3G15700	2.84	-0.35
AT5G17820	4.10	-0.54	AT1G65670	2.93	-0.35
AT4G25220	3.20	-0.54	AT3G49580	3.62	-0.35
AT2G46740	3.26	-0.54	AT1G01750	4.38	-0.35
AT4G40090	2.96	-0.53	AT3G55130	3.04	-0.35
AT2G43590	3.48	-0.53	AT1G44970	3.40	-0.35
AT2G26560	4.46	-0.52	AT3G51350	2.88	-0.35
AT5G20790	4.72	-0.52	AT2G28700	2.95	-0.35
AT3G09925	3.48	-0.52	AT1G17615	3.91	-0.35

AT2G43590	2.89	-0.52	AT4G25110	2.93	-0.35
AT1G02360	5.05	-0.51	AT5G01490	3.91	-0.35
AT3G03530	3.88	-0.51	AT5G48850	3.28	-0.35
AT3G30775	2.88	-0.51	AT1G74460	3.25	-0.35
AT5G62110	2.71	-0.50	AT1G51620	3.02	-0.35
AT2G24720	3.24	-0.50	AT3G52820	3.21	-0.35
AT1G78000	3.37	-0.49	AT1G08790	5.86	-0.35
AT5G26300	3.27	-0.49	AT5G59680	2.80	-0.34
AT3G16530	2.87	-0.49	AT5G22460	2.84	-0.34
AT1G01680	3.60	-0.48	AT1G55020	2.85	-0.34
AT4G30320	4.07	-0.48	AT4G26790	2.99	-0.34
AT1G30870	4.07	-0.47	AT4G26790	4.12	-0.34
AT4G09110	4.41	-0.46	AT5G50750	3.49	-0.34
AT1G66200	4.39	-0.46	AT1G04220	2.81	-0.33
AT5G39580	3.02	-0.46	AT1G08050	3.96	-0.33
AT1G11655	3.27	-0.46	AT3G04720	3.65	-0.33
AT3G48640	3.81	-0.46	AT5G53870	3.06	-0.33
AT5G24210	2.84	-0.46	AT1G53270	3.55	-0.33
AT1G24020	3.98	-0.46	AT3G19615	2.66	-0.33
AT5G54040	2.87	-0.46	AT1G51800	3.07	-0.33
AT3G60330	3.50	-0.45	AT2G28160	3.18	-0.33
AT4G16260	2.94	-0.45	AT1G70410	3.09	-0.33
AT3G12540	3.99	-0.45	AT4G24140	4.44	-0.33
AT1G34520	3.13	-0.45	AT4G25310	3.28	-0.33
AT4G32810	3.20	-0.45	AT1G54540	4.35	-0.33
AT3G49960	2.96	-0.45	AT3G12540	3.92	-0.33
AT2G25240	3.51	-0.45	AT4G12010	5.20	-0.32
AT1G32350	4.31	-0.45	AT5G58860	2.84	-0.32
AT2G31425	2.99	-0.44	AT1G05560	2.78	-0.32
AT5G16980	2.85	-0.44	AT3G24290	2.77	-0.32
AT3G13610	6.66	-0.44	AT2G36295	2.73	-0.32
AT4G29180	4.01	-0.44	AT3G62590	4.77	-0.32
AT1G02220	2.92	-0.44	AT5G65980	4.36	-0.32
AT3G10710	3.30	-0.44	AT5G64100	3.78	-0.32
AT5G43520	3.88	-0.43	AT2G43820	3.26	-0.32
AT4G28940	3.95	-0.43	AT4G30140	4.67	-0.32
AT3G48850	4.13	-0.43	AT5G13580	3.14	-0.32
AT1G48260	3.62	-0.43	AT1G71140	4.17	-0.31
AT5G25260	4.72	-0.43	AT1G78990	3.61	-0.31
AT4G16260	2.65	-0.43	AT2G33710	2.90	-0.31
AT1G66200	2.89	-0.42	AT4G18250	2.77	-0.31
AT1G60050	2.66	-0.42	AT5G59130	4.23	-0.31
AT3G46280	2.87	-0.42	AT5G06720	3.08	-0.31
AT1G51880	3.04	-0.42	AT5G49340	3.45	-0.31
AT4G33020	3.78	-0.42	AT2G26410	3.17	-0.31
AT3G16150	3.01	-0.42	AT4G35420	4.76	-0.31
AT1G24020	4.69	-0.42	AT4G25030	3.10	-0.31
AT5G42510	4.02	-0.42	AT3G63380	3.65	-0.31

AT1G24020	3.49	-0.42	AT4G16920	3.41	-0.31
AT2G20520	3.51	-0.42	AT2G23130	5.12	-0.31
AT4G10510	3.73	-0.41	AT1G57630	2.99	-0.31
AT4G36820	5.02	-0.41	AT2G44260	3.16	-0.31
AT5G24210	2.74	-0.41	AT4G24310	2.73	-0.31
AT5G39050	3.49	-0.41	AT1G29000	3.06	-0.31
AT1G13110	2.88	-0.41	AT1G56010	2.81	-0.31
AT2G21900	2.85	-0.41	AT3G62730	3.96	-0.30
AT1G74790	3.50	-0.41	AT3G51360	3.23	-0.30
AT3G44550	4.32	-0.41	AT5G20860	3.18	-0.30
AT1G02220	3.20	-0.41	AT2G18480	3.64	-0.30
AT5G49350	3.59	-0.41	AT4G25190	3.37	-0.30
AT3G14770	3.02	-0.41	AT2G41540	3.25	-0.30
AT3G22370	2.68	-0.41	At1G64790	2.84	1.19
AT1G74000	3.31	-0.40	AT1G66870	2.81	0.62
AT3G12700	4.31	-0.40	AT3G48740	2.72	0.58
AT5G20860	3.27	-0.40	AT3G27940	3.18	0.54
AT2G43510	3.24	-0.40	AT5G23660	2.77	0.53
AT4G16260	3.30	-0.40	AT4G08290	3.71	0.48
AT3G12700	3.36	-0.40	AT5G48110	3.23	0.45
AT2G20520	2.98	-0.40	AT2G22980	3.57	0.45
AT4G26790	4.44	-0.40	AT5G42200	6.13	0.42
AT3G46270	3.98	-0.40	AT5G45310	2.80	0.38
AT5G50260	2.74	-0.39	AT3G59480	3.18	0.38
AT5G19790	3.88	-0.39	AT5G42200	2.95	0.38
AT5G12420	4.56	-0.39	AT3G56210	2.78	0.37
AT4G13890	2.96	-0.39	AT1G74500	3.07	0.36
AT3G47780	2.89	-0.39	AT4G01330	3.34	0.36
AT1G48930	3.20	-0.39	AT2G14210	3.06	0.35
AT1G20180	3.95	-0.39	AT4G37260	3.36	0.35
AT4G02700	3.23	-0.38	AT4G07825	6.42	0.34
AT5G02230	5.73	-0.38	AT4G14270	2.66	0.32
AT5G46950	2.85	-0.38	AT2G22990	2.66	0.32
AT4G34930	2.77	-0.38	AT1G19610	3.20	0.32
			AT5G25620	3.13	0.31
