# Supplementary material for: The TIR-NB-LRR pair DSC1 and WRKY19 contributes to basal immunity of Arabidopsis to the root-knot nematode Meloidogyne incognita
Source: BMC Plant Biol. 2020 Feb 13;20:73. doi: 10.1186/s12870-020-2285-x (PMC7020509; doi:10.1186/s12870-020-2285-x)
Supplement: Supplementary file 5 — Additional file 5. Overview of primers used in qRT-PCR and for confirmation of T-DNA insert. [file 12870_2020_2285_MOESM5_ESM.pdf]

Additional file 5: Overview of primers used in RT-qPCR and for confirmation of T-DNA insert.

Identifier	Forward	Reverse
**Expression of**		
At4G12010 (DSC1)	TCGTGGGTCATGCTCGAATC	ACTCGGTATTTCAGTGGCCG
At4G12020 (WRKY19)	AATGTCCCTCTGGCGAACTC	CAGTACACCCAAGGCTCCAT
At4G12030 (BAT5)	CGAGTTGCGAATTTCCCACG	GCTCGTTTGAGTCAGATTCGG
At4G12040 (SAP7)	TCCGACTTTTCTGTTGAAGGTGA	CATGTTGGATGGTGACCCGA
At4G12050 (AHL26)	AAGATGAAGAACAAAATTCCAGCTT	CTAGCATGGAAAGGAGGAGGA
At5G60390 (EF1a)	GAGTACCACCTTTGGGACG	TTGGGTCCTTCTTGTCCACG
**T-DNA insert**		
DSC1 wildtype	TTAAGCGGAAACAACATCGAG	ACAACCTGGTTCTTCACCACC
dsc1-1 allele	ATTTTGCCGATTTCGGAAC	ACAACCTGGTTCTTCACCACC
WRKY19 wildtype	TTCATCAACAAGTTTGGCCTC	CCAATCATCATTCATAACCGG
wrky19-1 allele	ATTTTGCCGATTTCGGAAC	CCAATCATCATTCATAACCGG
BAT5 wildtype	TTCTTTCACATGGTTCAAGCC	ACAGCCGACCATAAACAACAG
bat5-2 allele	ATTTTGCCGATTTCGGAAC	ACAGCCGACCATAAACAACAG
