# Supplementary material for: The TIR-NB-LRR pair DSC1 and WRKY19 contributes to basal immunity of Arabidopsis to the root-knot nematode Meloidogyne incognita
Source: BMC Plant Biol. 2020 Feb 13;20:73. doi: 10.1186/s12870-020-2285-x (PMC7020509; doi:10.1186/s12870-020-2285-x)
Supplement: Supplementary file 6 — Additional file 6. Overview of primers used in qRT-PCR for a set of selected genes in Table 1 [file 12870_2020_2285_MOESM6_ESM.pdf]

Additional file 6: Overview of primers used in RT-qPCR for six selected genes from Table 1

Gene ID	Gene Description	WRKY domain	Primer Sequence
AT1G56280	drought-induced 19 (Di19)	No	For: GAGTTTGCTTGCCCGTTCTG<br>Rev: GGCATACCGCGTTCTTCGAC
AT5G06905	Cytochrome P450, family 93, subfamily D peptide 1	Yes	For: TCACGCTTCTCTGGTGTTGAG<br>Rev: AGCAGTCCCTGAAGCCGAA
AT5G06900	Cytochrome P450, family 712, subfamily A polypeptide 2	Yes	For: CAAGCTCTCAATCCGCTACGG<br>Rev: TGCATTGTGGGACGGTTCAG
AT5G45380	DEGRADATION OF UREA 3 (DUR3)	Yes	For: GCAGTATGGGGTTAGTGGACC<br>Rev:ACCGTGTGAGCATTAGGAGC
AT5G23660	Sugars Will Eventually be Exported Transporter 12 (SWEET 12)	No	For: CGATGCTTTGGCTCTACTACGC<br>Rev: GCGAAGGCAACAAAGATGGAG
AT4G26010	Peroxidase super family protein	Yes	For: GACTCGGTCGCATTAGCTGG<br>Rev: CGGAATTGTTGGTCCGGGTA
